# Safety profiles of sevoflurane in pediatric patients: a real-world pharmacovigilance assessment based on the FAERS database

**DOI:** 10.3389/fphar.2025.1548376

**Published:** 2025-02-10

**Authors:** Chuang Yang, Bangjian Deng, Qiang Wen, Pei Guo, Xiang Liu, Chen Wang

**Affiliations:** ^1^ Ministry of Education Key Laboratory of Child Development and Disorders, Chongqing Key Laboratory of Pediatric Metabolism and Inflammatory Diseases, Department of Pharmacy, Children’s Hospital of Chongqing Medical University, National Clinical Research Center for Child Health and Disorders, Chongqing, China; ^2^ Department of Clinical Pharmacy, The First Affiliated Hospital of Chongqing Medical and pharmaceutical College, The Sixth People’s Hospital of Chongqing, Chongqing Prevention and Treatment Center for Occupational Diseases, Chongqing, China

**Keywords:** FAERS, adverse events, pediatrics, sevoflurane, pharmacovigilance

## Abstract

**Objective:**

This study aimed to evaluate the safety profile of sevoflurane in pediatric populations using real-world data.

**Methods:**

Data were extracted from the Food and Drug Administration Adverse Event Reporting System (FAERS) from the first quarter of 2004 to the third quarter of 2024. We analyzed reports where sevoflurane was the primary suspect in individuals aged 0–18, employing disproportionality analysis to detect adverse events associated with sevoflurane. We also compared the adverse events related to sevoflurane between pediatric and adult populations.

**Results:**

The FAERS database yielded 21,838,627 adverse event reports for children, with 474 involving sevoflurane as the primary suspect. Descriptive analysis revealed a majority of reports from male patients, primarily reported by physicians. Disproportionality analysis identified significant System Organ Classes (SOC) signals associated with sevoflurane, meeting four detection criteria, including “Cardiac disorders,” “Respiratory, thoracic, and mediastinal disorders,” and “Vascular disorders.” The study also identified previously unreported adverse events, such as “Encephalopathy” and “Hypercapnia.” Notable differences in signals were observed between children and adults for “Pulmonary alveolar hemorrhage,” “Anaphylactic shock,” and “Hypotension.”

**Conclusion:**

Our analysis of the FAERS database identified several significant adverse events associated with sevoflurane in pediatrics, affecting the cardiovascular, respiratory, and nervous systems. Differences in adverse event signals between children and adults were also observed. Furthermore, the new adverse events (such as encephalopathy and hypercapnia) indicated that anesthesiologists should be more vigilant in administering sevoflurane.

## 1 Introduction

Sevoflurane, a widely utilized inhalation anesthetic, occupies a significant role in neonatal and pediatric anesthesia due to its rapid onset and lack of odor (Eger, 1994). Initially synthesized in the 1960s, sevoflurane gained Food and Drug Administration (FDA) approval for clinical use in 1995 (Wallin et al., 1975). It has largely replaced halothane, a previously widely used inhalational anesthetic, owing to its superior hemodynamic stability and reduced irritation to the respiratory tract (Doi and Ikeda, 1993; Friesen, 2024). Moreover, sevoflurane has demonstrated additional clinical benefits. Earlier studies suggest that sevoflurane can mitigate anxiety and distress in patients undergoing prolonged mechanical ventilation, and it can expedite recovery and extubation times post-administration (Pavcnik and Groselj Grenc, 2019). A randomized clinical trial also indicated that sevoflurane provides short-term analgesia and alleviates pain-related symptoms (Wang et al., 2022). However, the widespread use of sevoflurane in pediatrics has also raised concerns about its associated adverse reactions.

As an anesthetic, sevoflurane may exert potentially detrimental effects on the pediatric nervous system under specific conditions. Evidence from animal experiments indicates that prolonged or repeated exposure to sevoflurane postnatally can result in behavioral anomalies and cognitive deficits (Vutskits and Xie, 2016). Tang et al.’s research further demonstrated that neonatal mice exposed to sevoflurane exhibited significant cognitive impairments (Tang et al., 2020). Studies involving children have also suggested a correlation between sevoflurane exposure and an elevated risk of impaired motor function and reduced social competence scores (Walkden et al., 2020). Beyond neurological complications, sevoflurane has been implicated in inducing malignant hyperthermia in pediatric patients (Scholz, 1998). A case report by Shutes et al. identified that sevoflurane can induce hypercapnia in critically ill children (Shutes et al., 2017). Additionally, sevoflurane may provoke coughing, laryngospasms, agitation, and excitement in children. Nevertheless, there is a paucity of large-scale, real-world studies examining the safety profile of sevoflurane in pediatric populations. Consequently, it is imperative to explore the potential adverse effects of sevoflurane in children, with particular attention to previously unreported adverse events.

Real-world data are crucial for pharmacovigilance and adverse drug reaction research. Since 2004, the FDA’s Adverse Event Reporting System (FAERS) has amassed a substantial collection of real-world reports of adverse event related to drugs, which are instrumental in identifying and monitoring potential drug-related adverse events. As a self-reporting system, the FAERS database is updated quarterly and is freely available to the public. Consequently, this study aims to leverage the FAERS database for pharmacovigilance analysis on pediatric sevoflurane adverse event reports, thereby providing a valuable reference for the safe clinical application of sevoflurane in children.

## 2 Methods

### 2.1 Study design and data sources

In this retrospective pharmacovigilance study, adverse events were extracted from the FAERS database spanning from the first quarter of 2004 to the third quarter of 2024. The FAERS database comprises six data sets in ASCII format: demographic and administrative information (DEMO), drug information (DRUG), therapy start dates and end dates for reported drugs (THER), coded for the adverse events (REAC), patient outcomes for the event (OUTC), and indications for use/diagnosis (INDI).

Initially, we adhered to FDA guidelines to eliminate duplicate data. In DEMO, the FDA assigned CASEID, FDA_DT (date of FDA acceptance of the report), PRIMARYID (report unique identification number) for each report. When CASEID were identical in the DEMO data table, we retained cases with the most recent FDA_DT. Furthermore, when both CASEID and FDA_DT were identical, we selected cases with the most recent PRIMARYID.

**FIGURE 1:**
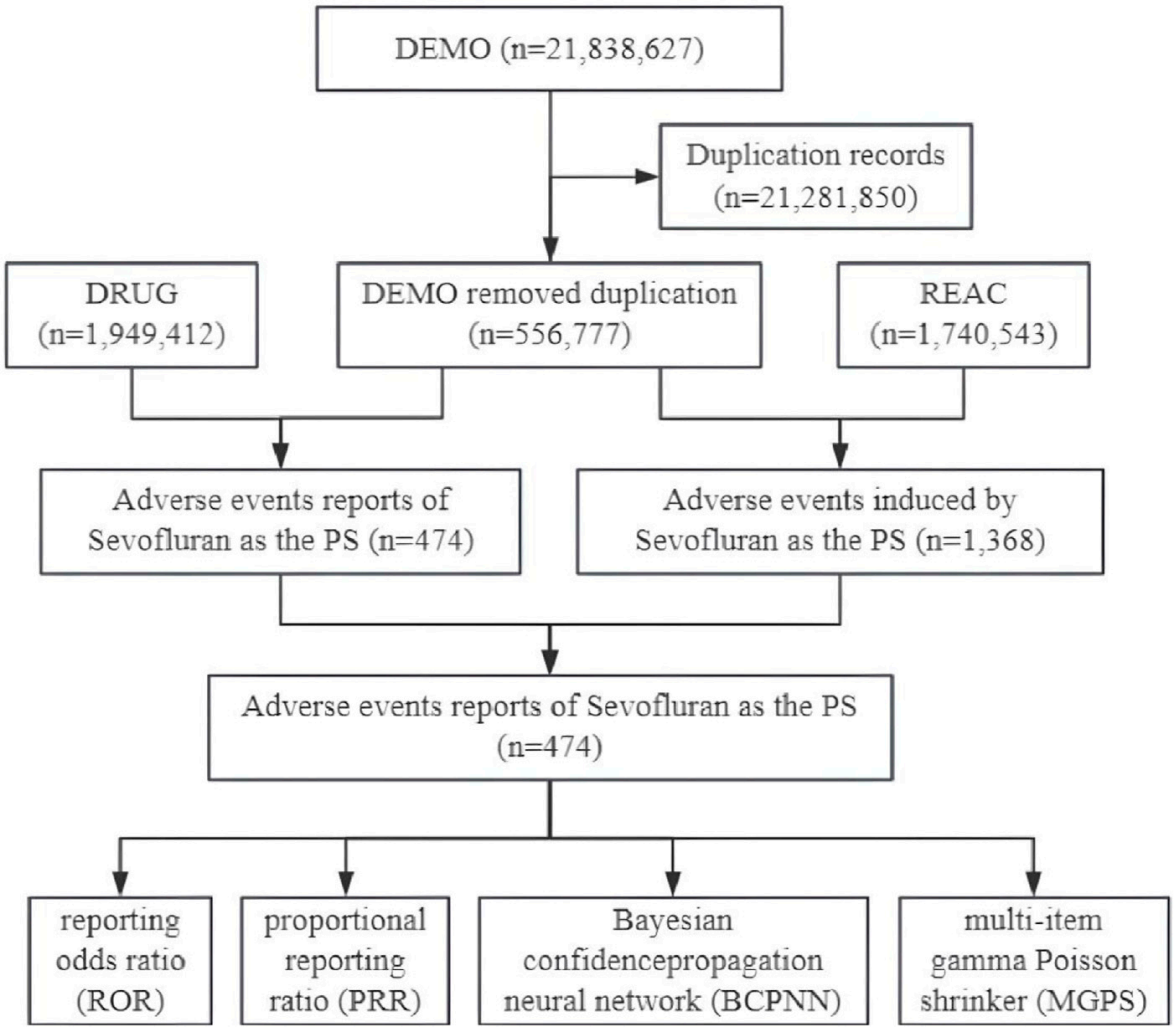
Data collection flow chart for adverse events of sevoflurane in children. DEMO, demographic and administrative information; DRUG, drug information; REAC, coded for the adverse events; PS, primary suspect.

Subsequently, we identified eligible reports based on drug names, encompassing both generic and brand names such as “SEVOFLURANE,” “ULTANE,” and “SEVORANE.” We also restricted our analysis to children under 18 years of age. For the reports of adverse event associated with sevoflurane, we focused on those with a primary suspect (PS) role code, indicating a potential causal relationship with the adverse events (Wen et al., 2024). The adverse events were categorized according to the preferred term (PT) of the Medical Dictionary for Regulatory Activities (MedDRA) version 25.1, with each PT corresponding to a major System Organ Class (SOC) (Yan et al., 2024). The study’s design flow is depicted in Figure 1.

### 2.2 Statistical analysis

The signal detection methodology in this study is founded on a four-fold contingency table (Supplementary Table S1). We employed four disproportionality analysis methods: reporting odds ratio (ROR), proportional reporting ratio (PRR), Bayesian confidence propagation neural network (BCPNN), and multi-item gamma Poisson shrinker (MGPS) (Yin et al., 2022; Zou et al., 2024). The formulas for these four detection methods and the criteria for positivity are detailed in Supplementary Table S2. For an adverse event signal to be considered in this study, it must satisfy the thresholds set by all four analytical algorithms, suggesting a potential link between the drug and adverse events (Jiang et al., 2024).

Furthermore, to investigate the disparity in adverse event signals between pediatric and adult populations exposed to sevoflurane, we utilized the ROR method and Fisher’s exact test (Hu et al., 2024). We collected a dataset of sevoflurane-related reports of adverse events, which contains only adults. After the same treatment, the PT signal that was identified as positive was retained and matched with the result in the child. The ROR used in this pharmacovigilance context differs slightly from its epidemiological counterpart, with the ROR algorithm’s four-fold table presented in Supplementary Table S3. The criteria for ascertaining signal differences for adverse events based on ROR values and Fisher’s exact test are outlined in Supplementary Table S4. All data processing and visualization were conducted using R software (version 4.0.0). The statistical significance was defined as *P* < 0.05.

## 3 Results

### 3.1 Descriptive analysis of clinical characteristics

**FIGURE 2:**
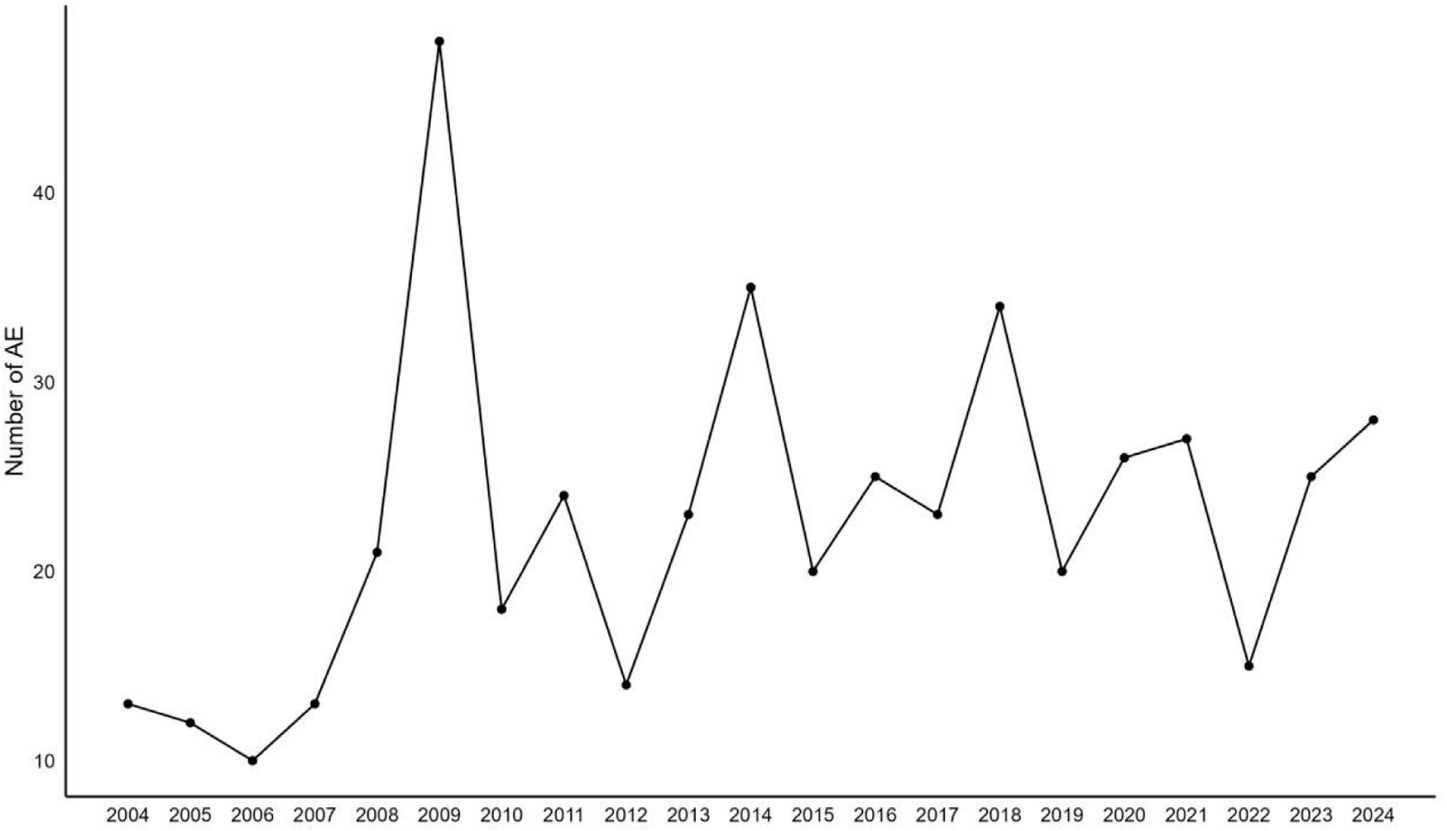
Distribution of number of adverse events over the reported years.

As depicted in Figure 1, out of a total of 21,838,627 reports in the DEMO dataset, we identified 556,777 unique reports after excluding 21,281,850 duplicates. Within this dataset, 474 cases involved sevoflurane as the primary suspect (PS), and 1,368 adverse event reports were recorded. Table 1 shows that males were more frequently represented (n = 238, 50.21%), whereas the female proportion was 36.7% (n = 174). The median age was 6, with 53.38% of the children being under 7 years old (n = 253). Physicians were the primary reporters of the cases (n = 241, 50.84%), and pharmacists reported only 30 cases (6.33%). The top three primary indications were all related to anesthesia. Hospitalization or prolonged hospital stays constituted the largest proportion of outcomes (n = 121, 25.53%). The top three reporting countries were the United States (n = 87, 18.35%), France (n = 52, 10.97%), and Russia (n = 31, 6.54%). The distribution of reporting years peaked in 2009, as shown in Figure 2.

**TABLE 1:** Clinical characteristics of reports with sevoflurane in children below 18 years old.

Characteristic	Counts (%)
Number of events	474
Sex	
Female	174 (36.71%)
Male	238 (50.21%)
Missing	62 (13.08%)
Age (years)	
<7	253 (53.38%)
≥7 and <18	221 (46.62%)
Median	6
Type of reporter	
Physician	241 (50.84%)
Pharmacist	30 (6.33%)
Other health-professional	111 (23.42%)
Consumer	51 (10.76%)
Missing	41 (8.65%)
Indications (TOP three)	
Anesthesia	103 (21.73%)
General anesthesia	81 (17.09%)
Induction of anaesthesia	76 (16.03%)
Outcome	
Death	59 (12.03%)
Life-threatening	70 (14.77%)
Hospitalization - initial or prolonged	121 (25.53%)
Disability	3 (0.63%)
Other serious outcome	221 (46.62%)
Reported countries (TOP three)	
US	87 (18.35%)
France	52 (10.97%)
Russia	31 (6.54%)

### 3.2 Descriptive analysis of concomitant drugs

**FIGURE 3:**
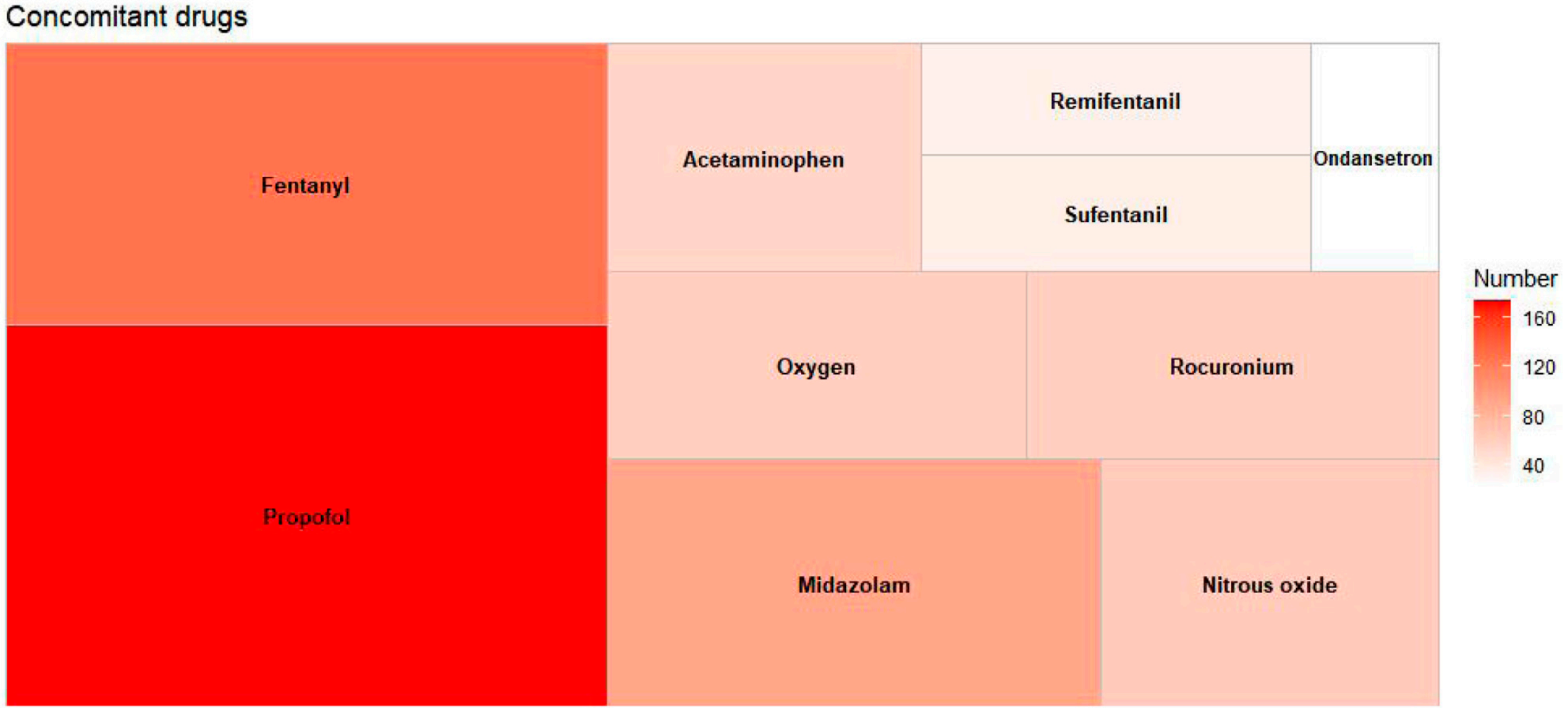
Top 10 co-reported concomitant drugs in adverse event reports.

Among the extracted adverse event reports, a total of 1,714 concomitant medications were identified. Figure 3 displays the top 10 concomitant medications, with propofol being the most frequently used, representing 10.15% of the total (n = 174). Other frequently noted concomitant medications include fentanyl (n = 128, 7.47%), midazolam (n = 92, 5.37%), rocuronium (n = 59, 3.44%), and acetaminophen (n = 54, 3.15%), among others.

### 3.3 SOC distribution of adverse event signals

**FIGURE 4:**
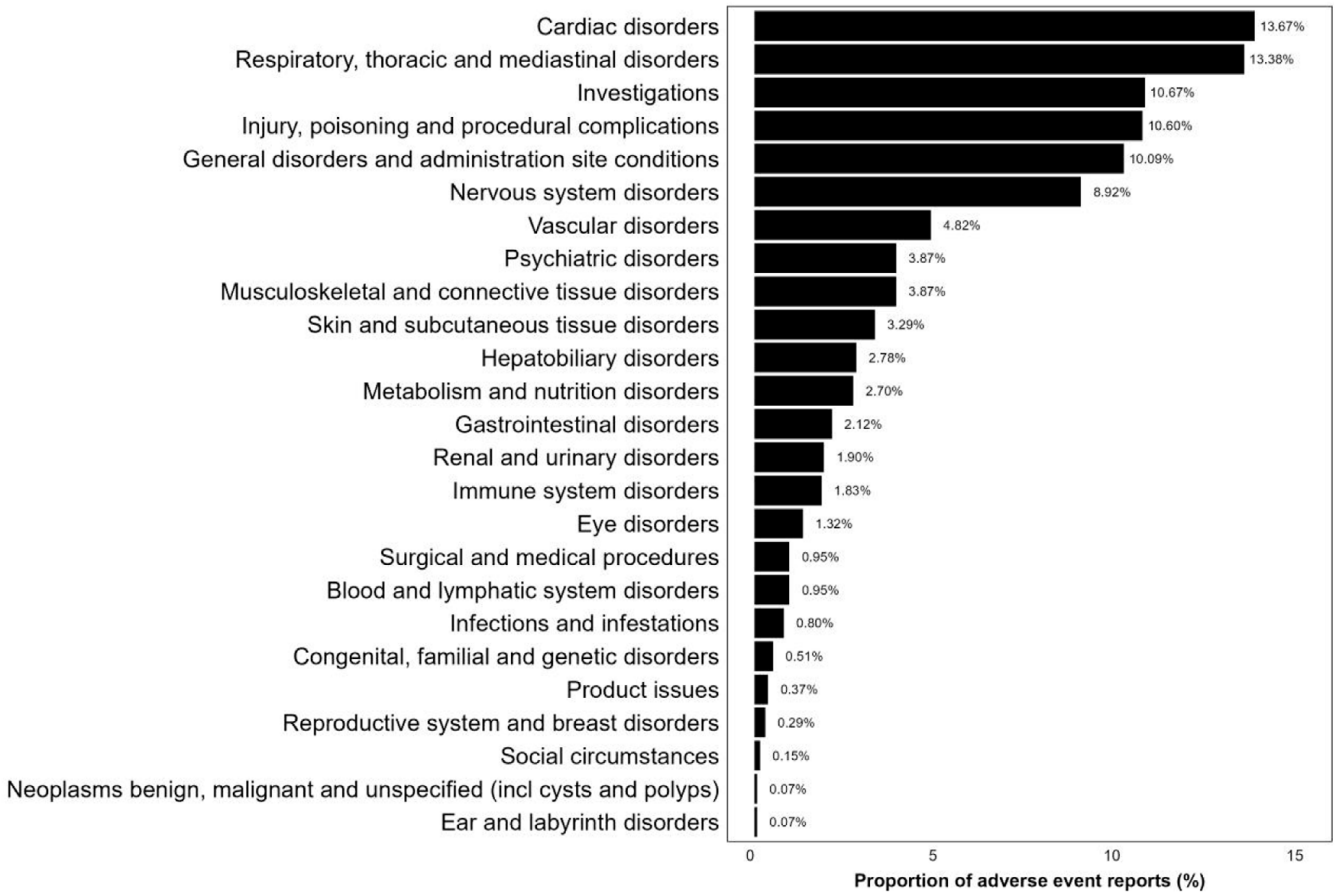
SOC distribution.

As shown in Figure 4, 1,368 reports of adverse event were classified using MedDRA, encompassing a total of 25 SOC signals. Among these, the three SOCs with the highest frequency were “Cardiac disorders” (13.67%), “Respiratory, thoracic, and mediastinal disorders” (13.38%), and “Investigations” (10.67%). Supplementary Table S5 provides a disproportionate analysis of signals at the SOC level, detailing the number of cases and signal strength. “Cardiac disorders,” “Respiratory, thoracic, and mediastinal disorders” and “Vascular disorders” satisfied all four criteria simultaneously. Additionally, the SOCs that met at least one test criterion were “Investigations,” “Musculoskeletal and connective tissue disorders,” and “Hepatobiliary disorders.”

### 3.4 PT distribution of adverse event signals

As shown in Table 2, a total of 81 positive signal categories were identified at the PT level, meeting the criteria of the four disproportionality analysis methods. Consistent with previous studies on sevoflurane, respiratory and cardiovascular adverse events are commonly reported. In this study’s findings, the most frequent PT signals in children were “Anaesthetic complication” (PT code: 10060938), “Cardiac arrest” (PT code: 10007515), “Malignant hyperthermia” (PT code: 10020844), and “Tachycardia” (PT code: 10043071), aligning with previous clinical trial outcomes. Notably, we also identified several significant adverse events not mentioned in the product instructions, including “Agitation” (PT code: 10001497), “Chills” (PT code: 10008531), “Pulmonary alveolar hemorrhage” (PT code: 10037313), “Hypercapnia” (PT code: 10020591), “Pulse absent” (PT code: 10037469), “Dystonia” (PT code: 10013983), and “Encephalopathy” (PT code: 10014625), among others.

**TABLE 2:** Signal strength of adverse event reports of sevoflurane at the PT level.

SOC	PT	Case (n)	ROR (95% Cl)	PRR (χ^2^)	EBGM (EBGM05)	IC (IC025)
Cardiac disorders	Cardiac arrest	39	15.39 (11.17–21.2)	14.98 (503.72)	14.81 (11.33)	3.89 (3.42)
	Tachycardia	28	6.47 (4.45–9.41)	6.36 (126.19)	6.33 (4.62)	2.66 (2.12)
	Bradycardia	25	13.36 (8.97–19.88)	13.13 (277.7)	13.01 (9.33)	3.7 (3.13)
	Ventricular tachycardia	10	20.82 (11.12–38.98)	20.68 (184.3)	20.36 (12.05)	4.35 (3.47)
	Cardio-respiratory arrest	10	7.26 (3.89–13.55)	7.21 (53.28)	7.18 (4.26)	2.84 (1.97)
	Ventricular fibrillation	8	28.76 (14.24–58.07)	28.6 (208.38)	27.99 (15.54)	4.81 (3.83)
	Torsade de pointes	7	41.99 (19.74–89.32)	41.78 (269.76)	40.48 (21.52)	5.34 (4.3)
	Ventricular extrasystoles	6	25.33 (11.27–56.93)	25.22 (136.87)	24.75 (12.57)	4.63 (3.52)
	Supraventricular tachycardia	4	16.86 (6.28–45.28)	16.82 (58.73)	16.61 (7.27)	4.05 (2.75)
	Arrhythmia	4	5.39 (2.01–14.4)	5.37 (14.18)	5.35 (2.35)	2.42 (1.12)
	Nodal arrhythmia	3	105.68 (32.5–343.57)	105.45 (286.5)	97.41 (36.32)	6.61 (5.09)
	Cardiovascular disorder	3	16.47 (5.27–51.5)	16.43 (42.93)	16.24 (6.25)	4.02 (2.56)
Respiratory, thoracic and mediastinal disorders	Laryngospasm	17	126.62 (76.71–209.01)	125.06 (1904.23)	113.9 (74.89)	6.83 (6.12)
	Bronchospasm	16	23.27 (14.15–38.26)	23.01 (330.95)	22.61 (14.92)	4.5 (3.79)
	Pulmonary oedema	12	12.11 (6.84–21.43)	12.01 (120.1)	11.91 (7.39)	3.57 (2.77)
	Hypoxia	10	7.51 (4.03–14.02)	7.47 (55.72)	7.43 (4.41)	2.89 (2.02)
	Pulmonary alveolar haemorrhage	8	67.88 (33.26–138.5)	67.49 (497.51)	64.12 (35.3)	6 (5.01)
	Hypercapnia*	7	68.48 (31.95–146.77)	68.13 (439.42)	64.7 (34.19)	6.02 (4.96)
	Respiratory depression	7	9.29 (4.41–19.57)	9.25 (51.13)	9.19 (4.92)	3.2 (2.17)
	Respiratory arrest	7	5.75 (2.73–12.1)	5.72 (27.18)	5.7 (3.06)	2.51 (1.49)
	Acute pulmonary oedema	5	48.47 (19.81–118.58)	48.3 (223.09)	46.56 (22.02)	5.54 (4.33)
	Laryngeal oedema	4	24.06 (8.93–64.78)	23.99 (86.49)	23.56 (10.28)	4.56 (3.25)
	Stridor	4	17.81 (6.63–47.84)	17.76 (62.4)	17.53 (7.67)	4.13 (2.83)
	Pulmonary haemorrhage	4	7.4 (2.76–19.79)	7.38 (21.94)	7.34 (3.22)	2.88 (1.58)
	Non-cardiogenic pulmonary oedema	3	74.59 (23.25–239.29)	74.43 (205.27)	70.35 (26.53)	6.14 (4.64)
	Haemoptysis	3	6.51 (2.09–20.28)	6.5 (13.9)	6.47 (2.50)	2.69 (1.24)
Investigations	Oxygen saturation decreased	24	12.63 (8.42–18.95)	12.43 (250.17)	12.32 (8.77)	3.62 (3.04)
	Blood creatine phosphokinase increased	8	9.40 (4.68–18.88)	9.35 (59.23)	9.29 (5.18)	3.22 (2.25)
	Pulse absent*	7	32.97 (15.54–69.95)	32.81 (210.43)	32 (17.05)	5.00 (3.96)
	Electrocardiogram qt prolonged	7	4.47 (2.12–9.4)	4.45 (18.67)	4.44 (2.38)	2.15 (1.12)
	Body temperature increased	6	7.06 (3.16–15.77)	7.03 (30.88)	7.00 (3.57)	2.81 (1.71)
	Blood pressure decreased	6	5.21 (2.33–11.65)	5.2 (20.27)	5.18 (2.64)	2.37 (1.28)
	Carbon dioxide increased	4	115.37 (41.39–321.52)	115.03 (414.49)	105.53 (44.76)	6.72 (5.36)
	Blood calcium decreased*	3	18.11 (5.79–56.69)	18.08 (47.72)	17.84 (6.87)	4.16 (2.7)
	Heart rate decreased	3	6.46 (2.07–20.1)	6.44 (13.73)	6.42 (2.48)	2.68 (1.23)
Injury, poisoning and procedural complications	Anaesthetic complication	57	709.96 (512.22–984.04)	680.42 (25149.82)	442.83 (336.99)	8.79 (8.34)
	Delayed recovery from anaesthesia	11	241.92 (126.7–461.9)	239.98 (2200.57)	201.88 (117.51)	7.66 (6.75)
	Post procedural complication	11	35.88 (19.66–65.48)	35.6 (359.85)	34.65 (20.95)	5.11 (4.27)
	Procedural complication	8	47.8 (23.55–97.01)	47.52 (351.22)	45.84 (25.35)	5.52 (4.53)
	Endotracheal intubation complication	5	158.74 (62.56–402.83)	158.17 (694.13)	140.71 (64.55)	7.14 (5.88)
	Airway complication of anaesthesia	4	158.63 (56.02–449.16)	158.17 (555.3)	140.71 (58.9)	7.14 (5.76)
	Anaesthetic complication neurological	3	271.74 (78–946.66)	271.15 (664.96)	223.47 (78.65)	7.8 (6.21)
	Unwanted awareness during anaesthesia	3	122.72 (37.47–401.89)	122.45 (329.5)	111.74 (41.41)	6.8 (5.28)
	Procedural nausea	3	115.28 (35.31–376.34)	115.03 (310.87)	105.53 (39.21)	6.72 (5.2)
	Procedural vomiting	3	63.4 (19.86–202.41)	63.27 (175.1)	60.3 (22.83)	5.91 (4.43)
General disorders and administration site conditions	Malignant hyperthermia	33	310.04 (211.27–454.99)	302.58 (8005.87)	244.38 (177.29)	7.93 (7.39)
	Chills*	12	7.13 (4.03–12.61)	7.08 (62.37)	7.05 (4.37)	2.82 (2.01)
	Hyperthermia	6	15.22 (6.79–34.09)	15.15 (78.4)	14.99 (7.63)	3.91 (2.8)
	Brain death*	3	9.93 (3.18–30.97)	9.91 (23.86)	9.84 (3.8)	3.3 (1.85)
Nervous system disorders	Coma*	8	4.52 (2.25–9.06)	4.5 (21.69)	4.48 (2.5)	2.16 (1.2)
	Dystonia*	7	5.19 (2.47–10.92)	5.17 (23.46)	5.15 (2.76)	2.36 (1.34)
	Encephalopathy*	7	4.83 (2.29–10.16)	4.81 (21.06)	4.79 (2.57)	2.26 (1.24)
	Brain injury*	4	12.82 (4.78–34.37)	12.78 (43.01)	12.66 (5.55)	3.66 (2.36)
	Hypoxic-ischaemic encephalopathy*	3	20.02 (6.39–62.71)	19.98 (53.25)	19.68 (7.57)	4.3 (2.84)
	Cerebral ischaemia*	3	17.13 (5.48–53.6)	17.1 (44.87)	16.88 (6.5)	4.08 (2.62)
	Myoclonus*	3	5.67 (1.82–17.64)	5.66 (11.46)	5.64 (2.18)	2.49 (1.05)
Vascular disorders	Hypotension	25	5.24 (3.52–7.79)	5.16 (83.85)	5.15 (3.69)	2.36 (1.79)
	Cyanosis*	6	4.61 (2.06–10.29)	4.59 (16.8)	4.58 (2.34)	2.19 (1.10)
	Circulatory collapse	5	9.08 (3.76–21.92)	9.05 (35.57)	8.99 (4.3)	3.17 (1.98)
	Hypertensive crisis	3	25.88 (8.24–81.27)	25.82 (70.16)	25.33 (9.72)	4.66 (3.2)
	Hypovolaemic shock	3	23.63 (7.53–74.12)	23.58 (63.68)	23.16 (8.9)	4.53 (3.07)
	Haemodynamic instability	3	8.74 (2.81–27.25)	8.73 (20.39)	8.67 (3.35)	3.12 (1.67)
Psychiatric disorders	Agitation	14	3.23 (1.9–5.46)	3.2 (21.22)	3.2 (2.06)	1.68 (0.93)
	Staring	5	17.07 (7.05–41.31)	17.01 (74.35)	16.8 (8.02)	4.07 (2.88)
	Delirium	5	6.03 (2.5–14.53)	6.01 (20.79)	5.98 (2.87)	2.58 (1.4)
	Panic reaction	4	25 (9.28–67.36)	24.93 (90.13)	24.47 (10.68)	4.61 (3.30)
Musculoskeletal and connective tissue disorders	Rhabdomyolysis	22	22.35 (14.61–34.18)	22.01 (433.91)	21.65 (15.17)	4.44 (3.82)
	Muscle rigidity*	6	13.54 (6.05–30.32)	13.49 (68.65)	13.35 (6.8)	3.74 (2.64)
	Musculoskeletal disorder	3	30.68 (9.75–96.54)	30.61 (83.91)	29.91 (11.46)	4.9 (3.44)
	Trismus*	3	12.23 (3.92–38.18)	12.21 (30.57)	12.1 (4.67)	3.6 (2.14)
Hepatobiliary disorders	Acute hepatic failure	5	7.3 (3.02–17.6)	7.27 (26.91)	7.24 (3.46)	2.86 (1.67)
	Hepatotoxicity	5	6.23 (2.58–15.03)	6.21 (21.78)	6.19 (2.96)	2.63 (1.45)
	Hepatic failure	4	4.6 (1.72–12.28)	4.58 (11.18)	4.57 (2.01)	2.19 (0.90)
	Hepatorenal failure	3	131.18 (39.91–431.16)	130.9 (350.47)	118.72 (43.87)	6.89 (5.36)
	Hepatitis acute	3	23.34 (7.44–73.21)	23.29 (62.84)	22.89 (8.79)	4.52 (3.05)
Immune system disorders	Anaphylactic reaction	11	4.44 (2.45–8.04)	4.41 (28.96)	4.4 (2.67)	2.14 (1.30)
	Anaphylactic shock	9	12.12 (6.28–23.42)	12.05 (90.39)	11.95 (6.89)	3.58 (2.66)
Metabolism and nutrition disorders	Metabolic acidosis	10	6.18 (3.31–11.54)	6.15 (42.92)	6.12 (3.63)	2.61 (1.74)
	Hyperkalaemia	5	10.3 (4.27–24.89)	10.27 (41.52)	10.2 (4.88)	3.35 (2.16)
	Acidosis	4	8.98 (3.35–24.05)	8.96 (28.09)	8.90 (3.90)	3.15 (1.86)
	Lactic acidosis	4	4.85 (1.82–12.98)	4.84 (12.16)	4.83 (2.12)	2.27 (0.98)

*Adverse event, not recorded in the drug labels/datasheets; PT, preferred terms; ROR, reporting odds ratio; CI, confidence interval; PRR, proportional reporting ratio; χ^2^, chi-squared; EBGM05, lower limit of 95% confidence interval of EBGM; IC, information component; IC025, the lower limit of 95%CI, of the IC.

### 3.5 Signal differences of sevoflurane in children and adults

**FIGURE 5:**
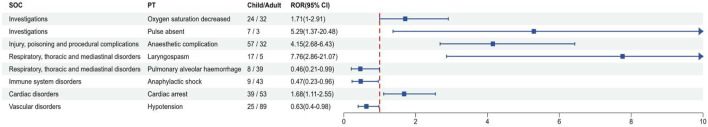
Signal differences of sevoflurane in children and adults. SOC, System organ class; PT, preferred terms; ROR, reporting odds ratio.

Figure 5 showed the difference in the adverse event signal of sevoflurane in adults and children. In comparison to adults, sevoflurane was more likely to cause “Pulmonary alveolar hemorrhage” in children (ROR: 0.46, 95% CI: 0.21–0.99, P = 0.04), “Anaphylactic shock” (ROR: 0.47, 95% CI: 0.23–0.96, P < 0.001), and “Hypotension” (ROR: 0.63, 95% CI: 0.40–0.98, P = 0.04). Conversely, high-risk adverse events in adults included “Pulse absent” (ROR: 5.29, 95% CI: 1.37–20.48, P = 0.01), “Anaesthetic complication” (ROR: 4.15, 95% CI: 2.68–6.43, P < 0.001), “Laryngospasm” (ROR: 7.76, 95% CI: 2.86–21.07, P < 0.001), and “Cardiac arrest” (ROR: 1.68, 95% CI: 1.11–2.55, P = 0.01). Supplementary Figure S1 and Supplementary Table S6 present the comparative results of adverse event signals between all children and adults.

## 4 Discussion

Since its introduction to the market, sevoflurane has been extensively utilized in pediatric anesthesia due to its rapid induction, lack of respiratory irritation, children’s ease in accepting its odor, and suitability for brief and outpatient surgical procedures. Generally, sevoflurane is regarded as a relatively safe inhalational anesthetic when compared to other anesthetic techniques. Nevertheless, numerous adverse drug reactions associated with sevoflurane have been documented in various clinical trials. Given the limitations in sample size within these trials, large-scale real-world data is indispensable for identifying potentially severe adverse events. This study compiled real-world adverse event data related to pediatric sevoflurane use, concentrating on events where sevoflurane was the PS, and analyzed novel and other significant adverse reactions to inform rational drug use in pediatric clinical practice.

In this study, reports of adverse event associated with sevoflurane were more prevalent in males than in females. A growing body of research suggests that there are sex-based differences in responses to anesthesia (Pleym et al., 2003; Kodaka et al., 2005; Buchanan et al., 2011). An earlier animal study indicated that male mice exhibit greater sensitivity to sevoflurane, and cognitive impairments induced by sevoflurane anesthesia were more pronounced in male rats (Zhang et al., 2022). While the mechanisms by which sexual dimorphism influences sevoflurane response remain unclear, these differences should be taken into account in clinical settings. Regarding the type of reporter, the majority of the reports originated from physicians and other healthcare professionals. Reports from healthcare professionals tend to be more consistent and accurately classified, facilitating the precise identification of adverse events (Wen et al., 2024). Concerning concomitant medications, our findings revealed that the primary medications co-administered were analgesics and sedatives commonly associated with pediatric anesthesia. The co-administration of propofol and sevoflurane can enhance the induction and maintenance of anesthesia, ensuring patients maintain a stable level of anesthesia throughout surgery. The combination of fentanyl and sevoflurane can synergistically improve anesthetic and analgesic effects, allowing for reduced dosages of each drug and minimizing the risk of adverse reactions (Fang et al., 2016). However, it is crucial to recognize that the risk of respiratory depression is significantly heightened when these drugs are combined.

A disproportionate analysis of adverse event signals retrieved from the FAERS database in this study revealed that SOC levels were most commonly found in “Cardiac disorders” and “Respiratory, thoracic and mediastinal disorders”. These adverse events encompass conditions such as “Tachycardia,” “Arrhythmia,” “Laryngospasm,” “Bronchospasm,” and others, aligning broadly with prior research. The cardiac effects of sevoflurane have been previously documented, including instances of rapid-onset bradycardia in children during sevoflurane-induced anesthesia (Walia et al., 2016). Kentaro et al. also described cases of transient cardiac arrest in children attributed to sevoflurane (Nogami et al., 2016). These findings underscore the need for vigilance regarding potential arrhythmias when sevoflurane is administered to children, to prevent severe cardiac adverse events. Regarding respiratory, thoracic, and mediastinal disorders, it is noteworthy that sevoflurane has been associated with pulmonary alveolar hemorrhage in children. Several previous case reports have detailed diffuse alveolar hemorrhage induced by sevoflurane (Mersh and Ross, 2018; Ahmed-Khan et al., 2023). While there is no definitive conclusion on sevoflurane’s causation of hemorrhage, existing studies suggest that volatile anesthetics can incite inflammation and lung endothelial damage by activating the arachidonic acid cascade (Fatheree and Leighton, 2004). We hypothesize that sevoflurane may induce alterations in alveolar permeability through a similar mechanism, precipitating alveolar hemorrhage. Further foundational research is warranted to validate this hypothesis.

The results of PT levels suggested that malignant hyperthermia is an adverse event with significant frequency and intensity in children. Malignant hyperthermia is a pharmacogenetic disorder characterized by a hypermetabolic response to sevoflurane (Rosenberg et al., 2015). Experimental evidence showed that malignant hyperthermia is related to the uncontrolled release of intracellular Ca+ ions in the sarcoplasmic reticulum of skeletal muscle, which leads to a series of symptoms such as increased oxygen consumption, ATP hydrolysis, and heat production (Jiang et al., 2008). Notably, pediatric cases of malignant hyperthermia constitute the majority of all reported cases (Strazis and Fox, 1993). There is a heightened susceptibility to malignant hyperthermia among children under sevoflurane anesthesia, potentially due to impairment of the thermoregulatory center (Rosenberg et al., 2007). Our findings, corroborated by existing evidence, highlight the need for vigilant core temperature monitoring during sevoflurane administration in children to preemptively avoid malignant hyperthermia.

Furthermore, we concentrated on adverse events not specified in the product information, particularly those that have not previously been reported. Hypercapnia, an adverse event not listed in the product instructions, has been previously associated with sevoflurane anesthesia in children (Machotta, 2002; Al-alami et al., 2009). Within the Investigations, we identified “Pulse absent,” which we hypothesize may correlate with the hypotension resulting from vasodilation post-sevoflurane anesthesia. Additionally, we detected encephalopathy within the nervous system. Sevoflurane has not been previously linked to encephalopathy. The occurrence of encephalopathy might be attributable to the neurotoxic effects of sevoflurane, which is recognized for its potential to induce cognitive impairment and neurodegenerative conditions (Tang et al., 2020). However, the precise mechanism by which sevoflurane might cause encephalopathy remains elusive, and further research is required to substantiate our observations. Consequently, we advocate for thorough safety assessments prior to administering sevoflurane anesthesia to children with pre-existing neurological disorders.

Our study represents the inaugural pharmacovigilance investigation of sevoflurane within a pediatric population. The research possesses several noteworthy strengths. Primarily, the FAERS database, which is the largest repository of post-marketing drug safety data, was utilized, offering an extensive array of real-world data for pharmacovigilance analyses. Additionally, our study identified novel and significant adverse events not previously aligned with the product specifications, including encephalopathy. We also observed disparities in adverse event profiles between adults and children, with pediatric patients exhibiting a heightened susceptibility to pulmonary alveolar hemorrhage, anaphylactic shock, and hypotension. It is our aspiration that these findings will serve as a valuable reference for the safe application of sevoflurane in pediatric anesthesia.

Nonetheless, we acknowledge the inherent limitations of our study. The FAERS database, being a spontaneous reporting system, is susceptible to underreporting, incomplete information, lack of causality, and other reporting irregularities. Second, sevoflurane dose was largely missing, so we did not consider the effect of dose, which is a key factor in adverse events. Thirdly, the data in the FAERS database lacked significant denominators to rule out existing cases, which also made it impossible to calculate the incidence of adverse events. Furthermore, the disproportionality analysis conducted in this study only suggests a statistical association between adverse events and the drug, lacking causality assessment.

## 5 Conclusion

Utilizing the FAERS database, this study extracted real-world safety data to assess the pediatric safety profile of sevoflurane. Adverse events of cardiac and respiratory nature, along with malignant hyperthermia, remain significant concerns in pediatric administration of sevoflurane. Furthermore, discrepancies in adverse event profiles between children and adults necessitate heightened vigilance in clinical practice.

## Data Availability

The original contributions presented in the study are included in the article/Supplementary Material, further inquiries can be directed to the corresponding author.
