# Supplementary material for: Safety profiles of sevoflurane in pediatric patients: a real-world pharmacovigilance assessment based on the FAERS database
Source: Front Pharmacol. 2025 Feb 10;16:1548376. doi: 10.3389/fphar.2025.1548376 (PMC11847804; doi:10.3389/fphar.2025.1548376)
Supplement: Supplementary file 1 [file DataSheet1.docx]

**Supplementary Table S1**. Fourfold table of disproportionality analysis.

|  | Reports with target adverse event | Reports with other adverse events |
| --- | --- | --- |
| Reports as the primary suspected drug | a | b |
| Reports with other drugs as the primary suspected drug | c | d |

a, number of reports containing both sevoflurane and target adverse event; b, number of reports containing other adverse events of sevoflurane; c, number of reports containing target adverse event of other drugs; d, number of reports containing other drugs and other adverse events

**Supplementary Table S2**. Formula and criteria of four algorithms for AE signal detection.

| Algorithm | Formula | Criteria |
| --- | --- | --- |
| ROR | ROR = $\frac{a/c}{b/d}$  95% CI = $e^{\ln(ROR)\pm1.96\sqrt{\frac{1}{a}+\frac{1}{b}+\frac{1}{c}+\frac{1}{d}}}$ | N ≥ 3,  Lower limit of 95% CI > 1 |
| PRR | PRR = $\frac{a}{a+b}/\frac{c}{c+d}$  χ^2^ = $\frac{[(ad-bc)^2](a+b+c+d)}{[(a+b)(c+d)(a+c)(b+d)}$ | PRR≥2, χ^2^≥4, N≥3 |
| BCPNN | IC = log_2_$\frac{a(a+b+c+d)}{(a+c)(a+b)}$  IC025 = $e^{\ln(IC)-1.96\sqrt{\frac{1}{a}+\frac{1}{b}+\frac{1}{c}+\frac{1}{d}}}$ | IC025 > 0 |
| MGPS | EBGM = $\frac{a(a+b+c+d)}{(a+c)(a+b)}$  EBGM05 = $e^{\ln(EBGM)-1.96\sqrt{\frac{1}{a}+\frac{1}{b}+\frac{1}{c}+\frac{1}{d}}}$ | EBGM05>2 |

N, number of reports; ROR, reporting odds ratio; CI, confidence interval; PRR, proportional reporting ratio; χ2, chi-squared; IC, information component; IC025, the lower limit of 95%CI of the IC; EBGM, empirical bayesian geometric mean; EBGM05, lower limit of 95% confidence interval of EBGM.

**Supplementary Table S3**. Fourfold table of disproportionality analysis for difference detection between child and adult.

|  | Reports with target adverse event | Reports with other adverse events |
| --- | --- | --- |
| Factor 1 | a | b |
| Factor 2 | c | d |

a, number of reports conforming to factor 1, containing the target adverse event with sevoflurane identified as the primary suspected drug; b, number of reports conforming to factor 1, containing other adverse events with sevoflurane identified as the primary suspected drug; c, number of reports conforming to factor 2, containing the target adverse event with sevoflurane identified as the primary suspected drug; d, number of reports conforming to factor 2, containing other adverse events with sevoflurane identified as the primary suspected drug.

**Supplementary Table S4**. Criteria of ROR and Fisher’s exact test for difference detection of sevoflurane signals.

|  | ROR | Fisher’s exact test |
| --- | --- | --- |
| Signals that factor 1 more likely to develop | ROR > 1, Lower limit of 95% CI > 1, N ≥ 3 | *P* < 0.05 |
| Signals that factor 2 more likely to develop | ROR<1, Upper limit of 95% CI<1, N≥3 | *P* < 0.05 |

ROR, reporting odds ratio.

**Supplementary Table S5**. Case number and signal strength of sevoflurane at the SOC level.

| SOC | Case(n) | ROR(95%Cl) | PRR(χ^2^) | EBGM(EBGM05) | IC(IC025) |
| --- | --- | --- | --- | --- | --- |
| Cardiac disorders | 187 | 8.59 ( 7.36 - 10.03 ) | 7.55 ( 1076.22 ) | 7.51 ( 6.6 ) | 2.91 ( 2.68 ) |
| Respiratory, thoracic and mediastinal disorders | 183 | 3.34 ( 2.86 - 3.90 ) | 3.03 ( 259.44 ) | 3.02 ( 2.65 ) | 1.6 ( 1.37 ) |
| Investigations* | 146 | 1.88 ( 1.58 - 2.23 ) | 1.78 ( 53.35 ) | 1.78 ( 1.54 ) | 0.83 ( 0.58 ) |
| Vascular disorders | 66 | 2.77 ( 2.16 - 3.54 ) | 2.68 ( 70.72 ) | 2.68 ( 2.18 ) | 1.42 ( 1.06 ) |
| Musculoskeletal and connective tissue disorders* | 53 | 1.53 ( 1.16 - 2.02 ) | 1.51 ( 9.40 ) | 1.51 ( 1.2 ) | 0.6 ( 0.19 ) |
| Hepatobiliary disorders* | 38 | 2.48 ( 1.80 - 3.43 ) | 2.44 ( 32.69 ) | 2.44 ( 1.86 ) | 1.29 ( 0.82 ) |

*represent at least meet one of the four algorithms. SOC, System organ class; ROR, reporting odds ratio; CI, confidence interval; PRR, proportional reporting ratio; χ2, chi-squared; EBGM, empirical bayesian geometric mean; EBGM05, lower limit of 95% confidence interval of EBGM; IC, information component; IC025, the lower limit of 95%CI of the IC.

**Supplementary Figure S**
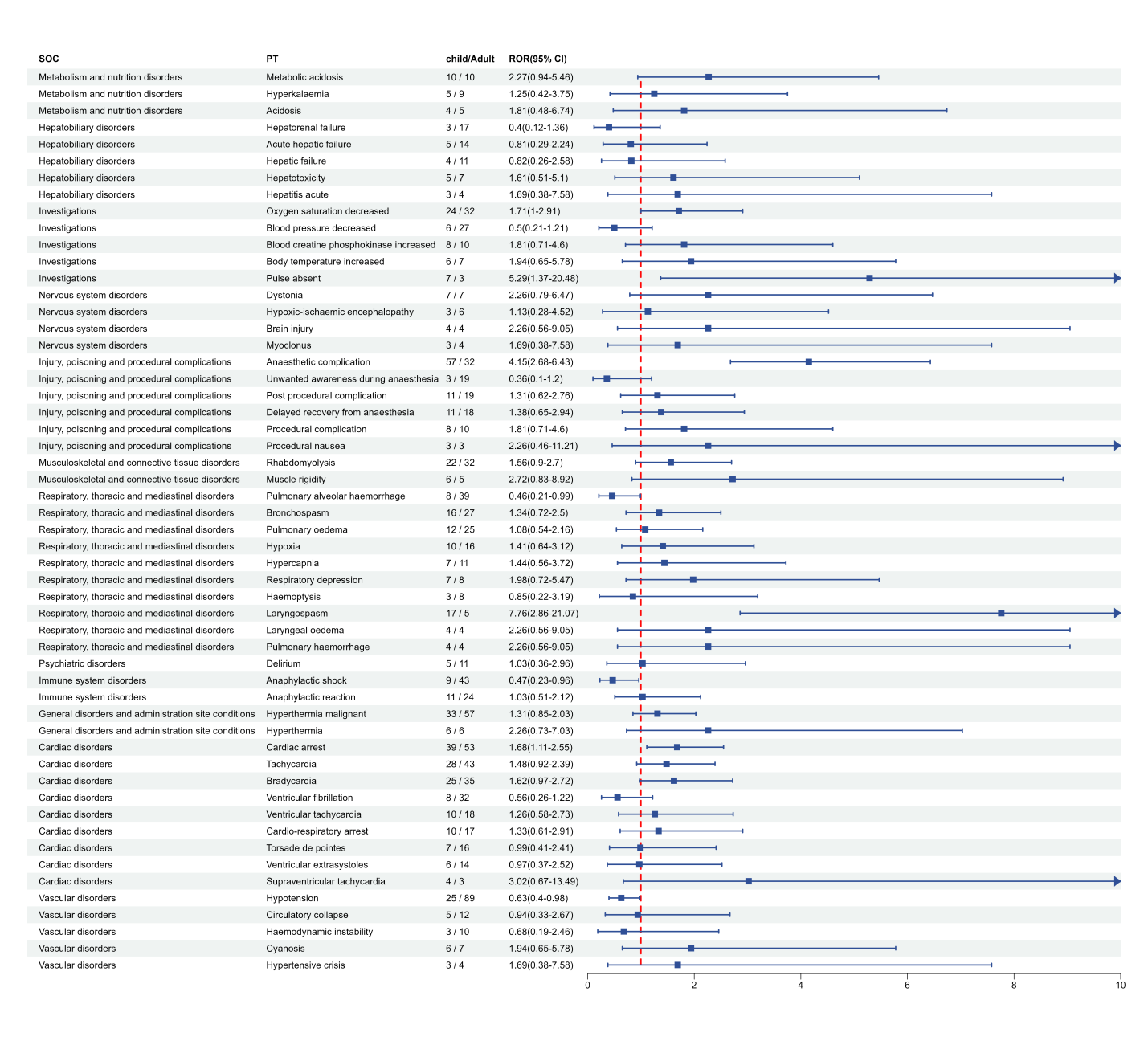
**1.** Differences in signal of sevoflurane between child and adult. SOC, System organ class; PT, preferred terms; ROR, reporting odds ratio.

**Supplementary Table S6**. Data of different signal of sevoflurane between child and adult.

| SOC | PT | a | b | c | d | ROR | RORL | RORU | *P* |
| --- | --- | --- | --- | --- | --- | --- | --- | --- | --- |
| Metabolism and nutrition disorders | Metabolic acidosis | 10 | 1358 | 10 | 3078 | 2.27 | 0.94 | 5.46 | 0.09 |
| Metabolism and nutrition disorders | Hyperkalaemia | 5 | 1363 | 9 | 3079 | 1.25 | 0.42 | 3.75 | 0.77 |
| Metabolism and nutrition disorders | Acidosis | 4 | 1364 | 5 | 3083 | 1.81 | 0.48 | 6.74 | 0.47 |
| Hepatobiliary disorders | Hepatorenal failure | 3 | 1365 | 17 | 3071 | 0.40 | 0.12 | 1.36 | 0.15 |
| Hepatobiliary disorders | Acute hepatic failure | 5 | 1363 | 14 | 3074 | 0.81 | 0.29 | 2.24 | 0.81 |
| Hepatobiliary disorders | Hepatic failure | 4 | 1364 | 11 | 3077 | 0.82 | 0.26 | 2.58 | 1.00 |
| Hepatobiliary disorders | Hepatotoxicity | 5 | 1363 | 7 | 3081 | 1.61 | 0.51 | 5.10 | 0.53 |
| Hepatobiliary disorders | Hepatitis acute | 3 | 1365 | 4 | 3084 | 1.69 | 0.38 | 7.58 | 0.45 |
| Investigations | Oxygen saturation decreased | 24 | 1344 | 32 | 3056 | 1.71 | 1.00 | 2.91 | 0.06 |
| Investigations | Blood pressure decreased | 6 | 1362 | 27 | 3061 | 0.50 | 0.21 | 1.21 | 0.13 |
| Investigations | Blood creatine phosphokinase increased | 8 | 1360 | 10 | 3078 | 1.81 | 0.71 | 4.60 | 0.21 |
| Investigations | Body temperature increased | 6 | 1362 | 7 | 3081 | 1.94 | 0.65 | 5.78 | 0.24 |
| Investigations | Pulse absent | 7 | 1361 | 3 | 3085 | 5.29 | 1.37 | 20.48 | 0.01 |
| Nervous system disorders | Dystonia | 7 | 1361 | 7 | 3081 | 2.26 | 0.79 | 6.47 | 0.15 |
| Nervous system disorders | Hypoxic-ischaemic encephalopathy | 3 | 1365 | 6 | 3082 | 1.13 | 0.28 | 4.52 | 1.00 |
| Nervous system disorders | Brain injury | 4 | 1364 | 4 | 3084 | 2.26 | 0.56 | 9.05 | 0.26 |
| Nervous system disorders | Myoclonus | 3 | 1365 | 4 | 3084 | 1.69 | 0.38 | 7.58 | 0.45 |
| Injury, poisoning and procedural complications | Anaesthetic complication | 57 | 1311 | 32 | 3056 | 4.15 | 2.68 | 6.43 | 0.00 |
| Injury, poisoning and procedural complications | Unwanted awareness during anaesthesia | 3 | 1365 | 19 | 3069 | 0.36 | 0.10 | 1.20 | 0.10 |
| Injury, poisoning and procedural complications | Post procedural complication | 11 | 1357 | 19 | 3069 | 1.31 | 0.62 | 2.76 | 0.55 |
| Injury, poisoning and procedural complications | Delayed recovery from anaesthesia | 11 | 1357 | 18 | 3070 | 1.38 | 0.65 | 2.94 | 0.42 |
| Injury, poisoning and procedural complications | Procedural complication | 8 | 1360 | 10 | 3078 | 1.81 | 0.71 | 4.60 | 0.21 |
| Injury, poisoning and procedural complications | Procedural nausea | 3 | 1365 | 3 | 3085 | 2.26 | 0.46 | 11.21 | 0.38 |
| Musculoskeletal and connective tissue disorders | Rhabdomyolysis | 22 | 1346 | 32 | 3056 | 1.56 | 0.90 | 2.70 | 0.14 |
| Musculoskeletal and connective tissue disorders | Muscle rigidity | 6 | 1362 | 5 | 3083 | 2.72 | 0.83 | 8.92 | 0.10 |
| Respiratory, thoracic and mediastinal disorders | Pulmonary alveolar haemorrhage | 8 | 1360 | 39 | 3049 | 0.46 | 0.21 | 0.99 | 0.04 |
| Respiratory, thoracic and mediastinal disorders | Bronchospasm | 16 | 1352 | 27 | 3061 | 1.34 | 0.72 | 2.50 | 0.41 |
| Respiratory, thoracic and mediastinal disorders | Pulmonary oedema | 12 | 1356 | 25 | 3063 | 1.08 | 0.54 | 2.16 | 0.86 |
| Respiratory, thoracic and mediastinal disorders | Hypoxia | 10 | 1358 | 16 | 3072 | 1.41 | 0.64 | 3.12 | 0.40 |
| Respiratory, thoracic and mediastinal disorders | Hypercapnia | 7 | 1361 | 11 | 3077 | 1.44 | 0.56 | 3.72 | 0.45 |
| Respiratory, thoracic and mediastinal disorders | Respiratory depression | 7 | 1361 | 8 | 3080 | 1.98 | 0.72 | 5.47 | 0.26 |
| Respiratory, thoracic and mediastinal disorders | Haemoptysis | 3 | 1365 | 8 | 3080 | 0.85 | 0.22 | 3.19 | 1.00 |
| Respiratory, thoracic and mediastinal disorders | Laryngospasm | 17 | 1351 | 5 | 3083 | 7.76 | 2.86 | 21.07 | 0.00 |
| Respiratory, thoracic and mediastinal disorders | Laryngeal oedema | 4 | 1364 | 4 | 3084 | 2.26 | 0.56 | 9.05 | 0.26 |
| Respiratory, thoracic and mediastinal disorders | Pulmonary haemorrhage | 4 | 1364 | 4 | 3084 | 2.26 | 0.56 | 9.05 | 0.26 |
| Psychiatric disorders | Delirium | 5 | 1363 | 11 | 3077 | 1.03 | 0.36 | 2.96 | 1.00 |
| Immune system disorders | Anaphylactic shock | 9 | 1359 | 43 | 3045 | 0.47 | 0.23 | 0.96 | 0.03 |
| Immune system disorders | Anaphylactic reaction | 11 | 1357 | 24 | 3064 | 1.03 | 0.51 | 2.12 | 1.00 |
| General disorders and administration site conditions | Hyperthermia malignant | 33 | 1335 | 57 | 3031 | 1.31 | 0.85 | 2.03 | 0.25 |
| General disorders and administration site conditions | Hyperthermia | 6 | 1362 | 6 | 3082 | 2.26 | 0.73 | 7.03 | 0.21 |
| Cardiac disorders | Cardiac arrest | 39 | 1329 | 53 | 3035 | 1.68 | 1.11 | 2.55 | 0.02 |
| Cardiac disorders | Tachycardia | 28 | 1340 | 43 | 3045 | 1.48 | 0.92 | 2.39 | 0.12 |
| Cardiac disorders | Bradycardia | 25 | 1343 | 35 | 3053 | 1.62 | 0.97 | 2.72 | 0.07 |
| Cardiac disorders | Ventricular fibrillation | 8 | 1360 | 32 | 3056 | 0.56 | 0.26 | 1.22 | 0.17 |
| Cardiac disorders | Ventricular tachycardia | 10 | 1358 | 18 | 3070 | 1.26 | 0.58 | 2.73 | 0.54 |
| Cardiac disorders | Cardio-respiratory arrest | 10 | 1358 | 17 | 3071 | 1.33 | 0.61 | 2.91 | 0.53 |
| Cardiac disorders | Torsade de pointes | 7 | 1361 | 16 | 3072 | 0.99 | 0.41 | 2.41 | 1.00 |
| Cardiac disorders | Ventricular extrasystoles | 6 | 1362 | 14 | 3074 | 0.97 | 0.37 | 2.52 | 1.00 |
| Cardiac disorders | Supraventricular tachycardia | 4 | 1364 | 3 | 3085 | 3.02 | 0.67 | 13.49 | 0.21 |
| Vascular disorders | Hypotension | 25 | 1343 | 89 | 2999 | 0.63 | 0.40 | 0.98 | 0.04 |
| Vascular disorders | Circulatory collapse | 5 | 1363 | 12 | 3076 | 0.94 | 0.33 | 2.67 | 1.00 |
| Vascular disorders | Haemodynamic instability | 3 | 1365 | 10 | 3078 | 0.68 | 0.19 | 2.46 | 0.77 |
| Vascular disorders | Cyanosis | 6 | 1362 | 7 | 3081 | 1.94 | 0.65 | 5.78 | 0.24 |
| Vascular disorders | Hypertensive crisis | 3 | 1365 | 4 | 3084 | 1.69 | 0.38 | 7.58 | 0.45 |

SOC, System organ class; PT, preferred terms; ROR, reporting odds ratio; RORL, the lower limit of 95%CI of the ROR; RORU, the upper limit of 95%CI of the ROR.
